# Tanycyte BMAL1 regulates high-fat diet weight gain and shapes arcuate neurogenesis in female mice

**DOI:** 10.1016/j.celrep.2026.117685

**Published:** 2026-07-13

**Authors:** Daniel Maxim Iascone, Pavel Pivarshev, Jianing Yang, Mariela Lopez Valencia, Sara B. Noya, Hongtong Lin, Corey D. Holman, Ron C. Anafi, Joseph L. Bedont, Amita Sehgal

**Affiliations:** 1 Howard Hughes Medical Institute, Chronobiology and Sleep Institute, Perelman School of Medicine, University of Pennsylvania, Philadelphia, PA, USA; 2 Institute for Diabetes, Obesity and Metabolism, Perelman School of Medicine, University of Pennsylvania, Philadelphia, PA, USA; 3 Department of Medicine, Chronobiology and Sleep Institute, Perelman School of Medicine, University of Pennsylvania, Philadelphia, PA, USA; 4 Present address: Department of Biological Sciences, Rensselaer Polytechnic Institute, Troy, NY, USA; 5 Present address: Department of Biological Sciences, Kent State University, Kent, OH, USA; 6 Lead contact

## Abstract

The hypothalamic radial-glia-like tanycyte population plays important and intertwined roles in metabolism, reproduction, and seasonality. Although these processes are circadian-regulated, the role of the molecular clock in tanycytes themselves has not yet been examined. We report that clock genes cycle with much higher amplitude in ventral tanycytes compared to more dorsal ependymocytes and that adult, tanycyte-specific knockout of core clock gene *Bmal1* reduces diet-associated weight gain and fat mass in female mice. Fate mapping studies show that female mice have higher baseline tanycyte-derived neurogenesis than males, with many of the resulting neurons localizing to the feeding-relevant arcuate nucleus. Female but not male mice show reduced tanycyte-derived arcuate neurogenesis after adult *Bmal1* deletion, with an increased proportion of newborn neurons acquiring a feeding-suppressing POMC neuropeptidergic fate. Together, our data support a role for tanycyte BMAL1 as a sex-specific regulator of body composition and hypothalamic adult neurogenesis.

## In brief

Iascone et al. show that tanycytes, specialized hypothalamic glia, demonstrate robust circadian rhythms and diet-responsive gene expression. Adult, tanycyte-specific knockout of core clock gene *Bmal1* inhibits tanycyte-derived arcuate nucleus neurogenesis in female but not male mice, and reduces weight gain and fat mass of female mice on a high-fat diet.

## Graphical Abstract

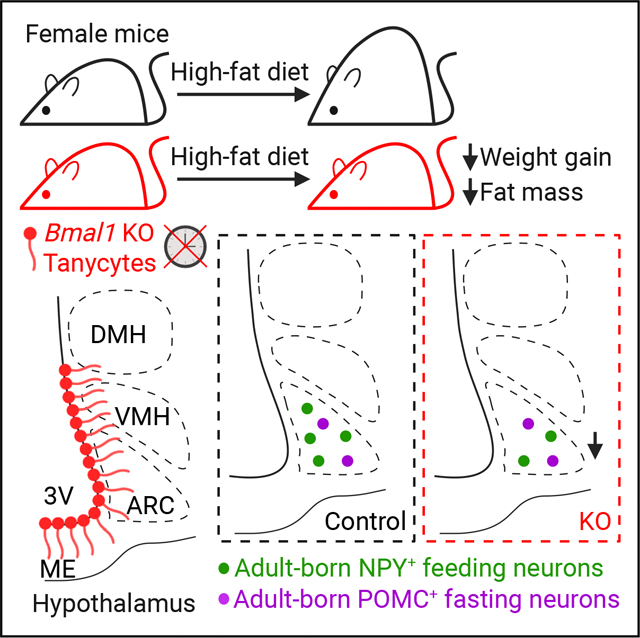

## INTRODUCTION

The circadian clock organizes the physiology and behavior of most lifeforms on Earth into 24-h rhythms aligned with the solar cycle. This organization begins in most single cells, where a molecular clock driven by an oscillatory transcription/translation feedback loop regulates tissue-specific clock-controlled gene expression throughout the daily cycle. Core mammalian clock components include the heterodimeric transcription factors BMAL1 and CLOCK, which cooperatively promote the expression of their own negative regulatory *Per* and *Cry* genes, ultimately leading to the inhibition of BMAL1:CLOCK driven transcription.^1^ At the circuit level, light input adjusts circadian phase in cells of the suprachiasmatic nucleus (SCN) in the brain, aligning clock-controlled genes that regulate SCN activity to the day:night cycle, which in turn orchestrates systemic autonomic, hormonal, and body temperature rhythms that entrain peripheral clocks to light.^2,3^ Ultimately, this organizes whole-body circadian rhythms in physiology and behavior, including sleep, feeding, and metabolism.

The health impacts of these rhythms are salient in modern society. Widely available artificial lighting and refrigeration cause mis-timed exposure of most humans to circadian entrainment cues such as light and food at evolutionarily unprecedented levels.^4^ Moreover, many people live on “flipped” circadian schedules, with >15% of the US workforce employed at shift work hours outside of a standard diurnal schedule.^5^ Elevated risk of obesity and metabolic disease in shift workers suggests not only direct health impacts from their extreme circadian misalignment,^6^ but also more subtle contributions of mis-timed circadian inputs to our society-wide epidemic of these conditions. This underscores the importance of understanding how the circadian clock is coupled to metabolism.

Such connections are widespread, with metabolism-relevant genes well represented among the ~15% of the mouse transcriptome under circadian control across tissues.^7,8^ One cell population of particular interest in linking circadian rhythms with metabolism is hypothalamic tanycytes. These radial glia-like cells line the floor of the 3rd ventricle (3V), adjacent to tuberal hypothalamic nuclei involved in feeding and metabolism: the ventromedial hypothalamus (VMH), arcuate nucleus (ARC), and median eminence (ME). Tanycytes play a number of roles in metabolism that are likely under some level of circadian control, including detection and import of peripheral metabolic hormones such as leptin, and gating of the central release of systemic TRH, CRH, and sex hormones.^9–12^

The evidence linking tanycytes to circadian clocks has thus far been indirect. The 3V possesses high-amplitude and photoperiod sensitive circadian rhythms,^13–15^ and tanycytes are critical downstream effectors of photoperiodically coded melatonin release, acting to coordinate breeding competence, feeding behavior, and metabolism in a seasonally adaptive manner.^16^ The SCN also signals to tanycytes to gate ARC access to peripheral sugars, thereby mediating circadian modulation of feeding and metabolism.^17^ But the tanycyte clock’s role in regulating feeding and metabolism has not previously been directly assessed, in part due to the difficulty of achieving its specific and efficient deletion.

In this study, we focus on the role of tanycytes as a source of new adult-born neurons known to modify the feeding circuitry in the ARC and ME in a diet-dependent manner.^18–23^ Analysis of published tanycyte transcriptomics led us to investigate a role of the circadian clock in hypothalamic tanycytes through RaxCreER-mediated adult deletion of the essential clock component *Bmal1*. While baseline rhythms in activity and sleep were unaltered, we observed a sex-specific reduction in weight and fat mass in female mice on a high-fat diet (HFD). This phenotype is likely due to a female-specific decrease in adult tanycyte-derived hypothalamic neurogenesis, which we found was associated with increased anorexigenic POMC^+^ fate among adult-born ARC neurons. Together, this suggests that tanycyte clock protein BMAL1 regulates weight and body composition homeostasis by influencing adult tanycyte neurogenesis.

## RESULTS

### Tanycytes but not ependymocytes show diet-induced expression changes in putative neurogenesis and circadian clock-relevant genes

Our interest in the tanycyte clock was prompted by possible links between clock genes and tanycyte neurogenesis, suggested by mining existing datasets.^24^ Campbell et al. fed mice either HFD or a low-fat diet for one week, and performed single-cell RNA sequencing of hypothalamic cell types including 3V tanycytes and neighboring ependymocytes. Tanycytes (but not less-specialized ependymocytes) showed robust diet-driven changes in gene expression (Figures 1A and 1B).

Importantly, our top hits also strongly suggested links to the circadian clock. EGR1 drives liver *Per1* expression and is rhythmically expressed with specific sensitivity to food reward in the brain,^34,35^ while Necdin binds directly to BMAL1 and promotes its stability.^36^ Together, these data suggested a potentially important role for the circadian clock in modulating tanycyte neurogenic responses to dietary fats, perhaps by intervening in tanycyte neurogenesis.

### Tanycyte circadian rhythms are ventrally enriched and susceptible to deletion by adult RaxCreER activation

Hypothalamic tanycytes have 4 subpopulations: α1/2 and β1/2. Briefly, β tanycytes line the ME and play prominent roles in blood-brain barrier function, peripheral nutrient sensing, and gating hormone release, while α tanycytes line the ARC and VMH and facilitate cross-talk of these nuclei with the cerebrospinal fluid.^37^ The remaining dorsal extent of 3V is largely composed of non-tanycyte ependymocytes (Figure 1D). While circumstantial evidence in the literature suggests clock gene enrichment in ventral β and perhaps α2 tanycyte populations, to our knowledge the relative contributions of 3V sub-populations have not previously been directly quantified.^13–15,38^ We tested this by sampling *Bmal1* and *Per2* clock gene transcripts by *in situ* hybridization in anatomically defined β tanycytes (ME-adjacent), α tanycytes (ARC/VMH-adjacent), and non-tanycyte ependymocytes (remainder of 3V) across circadian time in wild-type mice. As expected, *Bmal1* and *Per2* rhythms were dorsoventrally patterned, with the most robust rhythmicity in β tanycytes (Figures 1D and 1E).

These data supported targeting tanycyte *Bmal1* with RaxCreER^T2^, which is primarily expressed in ARC/ME-adjacent β and α2 tanycytes when activated in adulthood.^39,40^ We tested several tamoxifen (TAM) induction paradigms targeting the essential clock gene *Bmal1* in *RaxCreER*^*/*+^*;Bmal1^lox/lox^* and *RaxCreER*^*/*+^*;Bmal1*^*lox/*−^ mice (data not shown), and found that a 5-week induction with 250mg/kg TAM chow on a *Bmal1^lox/null^* background was needed to consistently reduce tanycyte BMAL1 expression and blunt the tanycyte cell-autonomous clock in adulthood (Figures S1A and S1B). The genetic combination of *RaxCreER* and *Bmal1^lox/null^* will hereafter be referred to as tanycyte *Bmal1* knockout mice (Tan^*Bmal1* KO^; red group in figures). At least 4 weeks were allowed on control chow after TAM treatment to mitigate direct TAM effects on behavior and physiology.

### Loss of tanycyte *Bmal1* sex-specifically reduces weight and fat mass on high-fat diet (HFD) in female mice

Because tanycyte neurogenesis has consistently been linked to weight gain, feeding, and metabolism,^18–23^ we tested whether tanycyte *Bmal1* knockout affected weight gain on HFD. Control *Bmal1*^*lox/*+^ or experimental Tan^*Bmal1* KO^ mice pre-treated with either CreER-activating TAM diet or control 2016 diet were subsequently fed on HFD for 12 weeks. Neither female nor male mice exhibited main effects of genotype or previous TAM/control treatment, nor did they exhibit significant interaction between these factors that would have indicated a specific effect of tanycyte *Bmal1* knockout. However, we were intrigued by an apparent trend in females: TAM treatment appeared to increase weight gain in genetic control mice while reducing weight gain in Tan^*Bmal1* KO^ mice (Figure S2A). In contrast, no such trend appeared in males (Figure S2B). In body composition analysis, both sexes had no significant effect on fat mass or lean mass following 12 weeks of HFD (Figures S2C and S2D).

To explore the possibility that loss of *Bmal1* in tanycytes might affect metabolic function in females, we performed indirect calorimetry in female Tan^*Bmal1* KO^ mice following 8 weeks of HFD, and observed that these mice exhibited lower weight and fat mass (but not lean mass) indicated by significant interactions between genotype and TAM treatment and consistent with the trend from our earlier experiments (Figure 2A). However, this change in body composition was not accompanied by significant differences in feeding, locomotion, energy expenditure, or respiratory exchange ratio (Figures 2B–2E). Separating these measurements by light period or dark period also did not reveal any significant differences in these metrics, though female Tan^*Bmal1* KO^ mice appear to drink less water during their nighttime active period (Figures S3I and S3J: significant interaction *p* < 0.05 repeated measures over time-of-day; *p* = 0.06 binned).

Notably, the differences in weight and fat mass we observed in female Tan^*Bmal1* KO^ mice after 8 weeks of HFD may not have been observable after 12 weeks due to a ceiling effect in weight gain by the genetic control group (Rax > *Bmal1* KO; gray dashed line in figures) that may have masked persistent differences in fat mass at earlier time points within the experimental window (Figure S2A). Overall, these results suggest that loss of tanycyte *Bmal1* in female mice attenuates weight gain on HFD through reduction in fat mass, but it is unclear which changes in underlying metabolic function drive these differences.

Importantly, given tanycytes’ known involvement in the seasonal regulation of activity,^16^ loss of tanycyte *Bmal1* did not alter the period or χ^2^ amplitude of wheel-running locomotor rhythms (Figures S3A and S3B). While the active period length of Tan^*Bmal1* KO^ mice was reduced in constant darkness, this was not associated with changes in wheel counts or bout number (Figures S4A–S4D). Since disturbances in sleep are frequently associated with metabolic dysregulation,^6,41^ we also tested for differences in sleep/wake behavior using the Piezo Sleep system. Neither the circadian timing nor total amount of sleep/wake was affected by tanycyte-specific *Bmal1* knockout in either sex (Figures S4E–S4H).

### Female mice have higher baseline adult tanycyte-derived ARC neurogenesis than males

From our sequencing analysis (Figures 1A and 1B), we suspected that tanycyte-derived ARC neurogenesis might contribute to attenuated weight gain on HFD in female Tan^*Bmal1* KO^ mice. But whether there is a baseline sex difference in tanycyte neurogenesis was unclear, with the two existing studies reporting either no sex effect^20^ or a weak trend toward increased female neurogenesis^42^ in the hypothalamus. Moreover, both studies lack lineage tracing and are susceptible to uneven incorporation of BrdU by dividing cells, leaving ambiguous what fraction of this neurogenesis is tanycyte-derived.

To fill this gap, we administered TAM chow to female and male *RaxCreER*^*/*+^*;Ai14*^*/*+^ reporter mice, permanently labeling tanycytes and their progeny with lox-stopped tdTomato in adulthood^43^ (Figure S1C). ~1-month after TAM induction, tdTomato-tagged ARC cells were then counted and categorized as neurons or astrocytes by morphology (Figure 3A). We observed higher baseline tanycyte-derived neurogenesis in female compared to male ARC using our system (Figures 3B and 3C). In contrast, tanycyte-derived ARC astrogenesis and neuron/astrocyte ratio had no sex difference.

### Tanycyte-derived adult ARC neurogenesis is sex-specifically reduced by adult *Bmal1* deletion in females, reflecting the depletion of adult-born orexinergic neurons

Having established this baseline, we next did similar lineage tracing in TAM-induced tanycyte *Bmal1*-deficient Tan^*Bmal1* KO^; *Ai14*^*/*+^ and genetic control *RaxCreER*^*/*+^*;Bmal1*^*lox/*+^*;Ai14*^*/*+^ ARC, in both females and males. Similarly to reduced weight gain we observed in these mice on HFD (Figure 2), adult tanycyte-derived neurogenesis was reduced in female but not male Tan^*Bmal1* KO^; *Ai14*^*/*+^ ARC (Figures 4A–4D). In contrast, adult tanycyte-derived astrogenesis was increased in both female and male Tan^*Bmal1* KO^; *Ai14*^*/*+^ mice, leading to a significantly reduced adult tanycyte-derived neuron/astrocyte ratio only in females (Figures 4C and 4D).

The ARC contains both orexinergic AgRP/NPY^+^ and anorexinergic POMC^+^ neuropeptidergic neurons that are specified at a late clock-competent stage, and there is considerable evidence for a circadian clock role in specifying cell fate.^38,44^ We hypothesized that reduced female-specific ARC neurogenesis caused by tanycyte *Bmal1* deletion (Figure 4) might reflect the selective depletion of adult-born orexinergic ARC neurons, potentially contributing to reduced female weight gain and fat mass on HFD (Figure 2). To investigate this possibility, we carried out lineage tracing in female mice with an extended 12-week chase on control chow (mirroring our HFD duration in Figure S2) and co-stained ARC for NPY and POMC. While there was no effect of tanycyte *Bmal1* deletion on the percentage of adult-born, tanycyte-derived NPY^+^ or NPY/POMC-negative ARC neurons, the proportion of adult-born POMC^+^ neurons was increased (Figures 4E and 4F). Together with decreased overall tanycyte neurogenesis, this is consistent with a skewing of tanycyte-derived ARC neuronal fate toward anorexigenic fate after adult tanycyte *Bmal1* deletion.

## DISCUSSION

In this work, we leveraged the *RaxCreER* driver to execute the first relatively specific *Bmal1* knockout in tanycytes *in vivo* and investigated the impact of this manipulation on diet-induced weight gain and hypothalamic adult neurogenesis. RaxCreER is well-characterized, and aside from hypothalamic tanycytes, targets only retinal Muller glia, cerebellum, and a handful of posterior pituicytes in adulthood.^39^ *Bmal1* deletion in these off-target cell-types is unlikely to drive the phenotypes we observe, not least because of attenuated HFD weight gain in female but not male mice after adult RaxCreER-mediated *Bmal1* deletion (Figure 2). This phenocopies female-specific attenuated HFD weight gain after focal irradiation-induced ablation of tanycyte neurogenic capacity.^20,21^ Thus, we interpret female-specific reduction of tanycyte-derived adult ARC neurogenesis after tanycyte *Bmal1* deletion (Figure 3) as a brake on female weight gain.

Consistent with this, *Bmal1* deletion shifts tanycyte-derived ARC neuron identities toward anorexigenic POMC^+^ fates (Figure 4). Importantly, blocking relatively small amounts of adult tanycyte neurogenesis is sufficient to influence weight gain in female mice on HFD,^20,21^ and adult-born POMC^+^ neurons can partially rescue feeding and metabolism on a *Pomc*-deficient background.^22^ This supports the potential functional relevance of the decreased tanycyte neurogenesis and anorexigenic fate shift we observe after adult tanycyte *Bmal1* deletion, even with relatively small absolute numbers of progeny neurons affected.

This model is coherent with the circadian clock’s role in mature ARC circuitry. While HFD only modestly impairs rhythmicity of ARC core clock genes, it dramatically weakens circadian rhythmicity of ARC peptides, together with overall decreased orexigenic (*Npy/Agrp/Hypocretin*) and increased anorexigenic (*Pomc/Cart*) mRNA expression.^45–47^ BMAL1 is also required for acute palmitate induced *Npy* expression in a hypothalamic cell line.^45^ In this context, skewing of adult-born neurons toward anorexigenic POMC^+^ fate after tanycyte *Bmal1* deletion (Figure 4) suggests a pro-orexigenic clock role across the developmental lifespan of female ARC neurons. Circadian BMAL1 competition for *cis* binding elements may contribute, as several other bHLH transcription factors promote anorexigenic fate during ARC development.^44^ It is also possible that migration and/or survival effects contribute to circadian regulation of adult-born orexigenic/anorexigenic ratios.

Importantly, our findings for tanycyte-derived ARC neurogenesis are consistent with previously studied circadian clock roles elsewhere, predominantly the hippocampus. Hippocampal neural stem cells divide on a circadian rhythm, hyper-proliferate in clock-deficient young animals, and hypo-proliferate in clock-deficient old animals.^48–51^ This likely reflects circadian clock roles in inhibiting excessive cell division and promoting self-renewal in young animals. In addition, BMAL1 has a potentially clock-independent role in promoting neural over glial daughter cell fate.^38^ Accordingly, sex-agnostic increased ARC astrogenesis after tanycyte *Bmal1*-deletion suggests that BMAL1-specific pro-neural effects are sex independent (Figure 4). Conversely, female-specific decreased ARC neurogenesis from *Bmal1*-deficient tanycytes suggests that BMAL1’s role in restraining excessive cell division and promoting self-renewal is sex-specific in tanycytes (Figure 4).

A hormonal interaction may contribute to sex-specific tanycyte responses to *Bmal1* deletion. Indeed, estrogens require tanycyte ERα to exert anorexigenic effects,^52^ which include reduced weight gain on HFD and regulation of ARC neurogenesis and adult-born cell fate in female mice.^53,54^ The circadian clock may gate tanycyte sensitivity to estrogen signaling via Per2 regulation of ERα degradation,^55^ CLOCK interactions with ERα,^56^ and/or other mechanisms.

### Limitations of the study

Altogether, we show that tanycytes, specialized hypothalamic glia, possess robust circadian rhythms and that adult, tanycyte-specific knockout of core clock gene *Bmal1* reduces diet-associated weight gain and tanycyte neurogenesis in female mice. However, we did not explicitly test functional integration of these neurons in the present study, nor did we directly parse the relative contributions of proliferation, specification, migration, and survival to the neurogenesis phenotypes we observe. We also did not observe a significant effect of tanycyte *Bmal1* deletion on food intake, leaving unclear whether a transient POMC-driven feeding reduction or some other factor underlies this effect. Nonetheless, the current observations are exciting given evidence for hypothalamic neurogenesis in humans,^57,58^ which may be susceptible to behavioral circadian interventions.

Finally, while we focus on ARC neurogenesis as one likely mechanism, we do not discount the possibility that other factors may also contribute to the regulation of female weight homeostasis by tanycyte BMAL1. Possibilities include the modulation of tanycytic gating of hormone release, modification of brain hormones, barrier functions gating peripheral signals, and/or active reception or transport of peripheral signals.^10–12,16,37^ And while parallels to the hippocampal neurogenesis literature suggest that our female-specific tanycyte neurogenesis effects reflect the disruption of the cell-autonomous circadian clock (see above), we cannot fully exclude possible non-circadian BMAL1 contributions to our female-specific tanycyte deletion phenotypes. This invites future exploration of the local clock’s role in tanycyte function, including well-documented roles in stress response, reproduction, photoperiodism, and more.^12,16,52^ Indeed, even behavioral outputs such as circadian locomotor rhythms and sleep behavior that were unaffected in our study under standard conditions (Figures S3 and S4) may well be regulated by the tanycyte clock in the context of seasonality or on different diets. Looking forward, we believe that the tanycyte clock’s impacts on physiology and behavior are a largely untapped field ripe for future study.

## RESOURCE AVAILABILITY

### Lead contact

Requests for further information and resources should be directed to and will be fulfilled by the Lead Contact, Amita Sehgal (amita@pennmedicine.upenn.edu).

### Materials availability

Materials generated in this study will be made available on request, but we may require a payment and/or a completed materials transfer agreement if there is potential for commercial application.

### Data and code availability

- Data reported in this paper will be shared by the lead contact upon request.
- This paper does not report original code.
- Any additional information required to reanalyze the data reported in this paper is available from the lead contact upon request.

## STAR★METHODS

### EXPERIMENTAL MODEL AND STUDY PARTICIPANT DETAILS

#### Animal housing, care, and genotyping

All animals were handled according to protocols approved by the Institutional Animal Care and Use Committee at University of Pennsylvania, Philadelphia (USDA registration 23-R-0023; AAALAC unit x 801; IACUC protocol 806387). Combinations of the following alleles were used for most studies: *RaxCreER*,^39^ *Bmal1* floxed allele (JAX #007668), *Bmal1* constitutive null allele (JAX #009100), and Ai14 fluorescent reporter (JAX #007914). Genetically modified mice were provided by Dr. Seth Blackshaw (*RaxCreER*) and Jackson Laboratories (other mutant lines). All lines not received on a C57BL6/J background were back-crossed at least 5 times to C57BL6/J, and wild-type C57BL6/J mice were used for the *in situ* hybridization (ISH) timecourse. Genotyping was conducted by Transnetyx, Inc using proprietary automated genotyping methods. Except where otherwise noted, mice were on a 12hr:12hr vivarium light:dark cycle.

### METHOD DETAILS

#### *In situ* hybridization: Clock gene circadian timecourse

Male mice were entrained to a 12hr:12hr light:dark cycle in custom circadian cabinets (Phenome Technologies), and released into constant darkness for 24hrs before beginning CT4-CT24 tissue collections on the second day in constant darkness, as previously described.^62,63^ Briefly, mice were sacrificed under dim red light, and their brains were collected fresh frozen in OCT (Tissue-Tek) and stored at −80C. 25 μm sections were collected on a Leica CM3050 cryostat, dry mounted on Superfrost Plus slides, and stained by chromogenic *in situ* hybridization with partially hydrolyzed riboprobes. Brain sections containing the tuberal hypothalamus were imaged on a ThermoFisher EVOS M7000 microscope, and ImageJ was used to quantify densitometry in anatomically defined β tanycyte-, α tanycyte-, and ependymocyte-rich portions of the 3rd ventricle. We then subtracted the intensity of nearby signal-poor tissue on each section to control for background color. At circadian expression troughs, low magnitude negative values were occasionally computed. For each probe, a scaling factor sufficient to raise the lowest negative value to 0 was added across the dataset to maintain relative differences among timepoints. 5 brain sections per mouse were quantified and averaged for each data point. Circadian analysis of ISH data was performed using the JTK_CYCLE algorithm within the MetaCycle R package.^59,60^

#### Riboprobes were generated from the following constructs

##### –Per2:

Accession #AI838843, PCR amplified with T3+T7 primers, riboprobe run off with T7 polymerase.

##### –Bmal1:

*Bmal1* Exon 8 was PCRed with primers (forward: ATGCAGAACACCAAGGAAGG, reverse: CTTCCTCGGTCACATCCTA), and cloned into pCRII-TOPO. PCR amplification was done with T7+Sp6 primers, and riboprobe was runoff with T7 polymerase.

#### Tamoxifen treatment

To induce Cre-dependent *Bmal1* deletion and tdTomato expression for lineage tracing, *RaxCreER*^*/*+^*;Bmal1^lox/null^;Ai14*^*/*+^ (Tan^*Bmal1* KO^) experimental and littermate control mice for all experiments were fed on one of the following pre-irradiated and vacuum-packed diets: recombination inducing red-dyed 250mg/kg tamoxifen diet (Inotiv/Envigo TD.130856) or control 2016 diet (Inotiv/Envigo 2916.cs) for 5 weeks, beginning at P30–40. Mice were weighed weekly during this time around mid-afternoon (~ZT7–10 on vivarium light cycle), and mice dropping more than 20% of their starting body weight were euthanized to prevent suffering. Except where otherwise noted, this was followed for all groups by 4 weeks on control 2016 diet, to allow recovery time for the tamoxifen-fed group before beginning other experimental manipulations.

#### High-fat diet treatment and MRI

Mice were weighed at the end of their recovery period after tamoxifen or control diet to establish a baseline weight, then moved to new cages with HFD (Inotiv/Envigo TD.06414: 60% calories from fat). Mice were then weighed weekly for 12 weeks, consistently around mid-afternoon (~ZT7–10 on vivarium light cycle). At the end of this time, mice were transferred to a satellite facility, where they underwent MRI measurement of fat:lean mass body composition on an EchoMRI-500 Body Composition Analyzer early in the morning (~ZT2–4 on vivarium light cycle). 1 mouse was excluded from weight gain and MRI analysis from the female “Control after TAM” group following data quantification after being identified as an outlier by a ROUT test (Q = 1%) of median weight gain measurements.^64^ A majority of mice from all experimental groups were then sacrificed at ZT12 for *Per2* ISH analysis.

#### Metabolic monitoring

Preclinical indirect calorimetry standards from the International Indirect Calorimetry Consensus Committee (IICCC) were followed for metabolic monitoring studies.^65^ Metabolic measurements in female mice following 8 weeks of HFD were acquired by indirect calorimetry using a Promethion CORE metabolic screening system (Sable Systems International). Mice were single housed for the duration of the experiment and had *ad libitum* access to HFD and water. Mice were acclimated to the metabolic cages for 48 h, followed by 48 h of data recording starting at 7:00 a.m. (lights on time). Immediately following the 48-h recording, mice were weighed and underwent body composition measurement for fat and lean mass (EchoMRI-500 Body Composition Analyzer). Oxygen consumption (VO2) and carbon dioxide production (VCO2) were measured every 5 min and used to calculate the respiratory exchange ratio (RER = VCO2/VO2) and energy expenditure using the Weir equation (Energy expenditure = 3.941 kcal/L × VO2 + 1.106 kcal/L × VCO2).^66^ Metabolic monitoring data was analyzed using CalR2 (https://calrapp.org/).^61^ Food and water intake along with body mass was continuously monitored gravimetrically using built-in hopper load cells. 1–4 mice per experimental group were excluded from food intake analysis prior to data quantification based on experimenter-observed food caching behavior during metabolic monitoring.^65^ 1 mouse was excluded from EE and RER analysis from the “Control” group (no TAM) following data quantification after being identified as an outlier by a ROUT test (Q = 1%) of VO_2_ measurements.

#### Wheel-running recordings

Wheel-running activity was measured as previously described.^67^ Briefly, mice were transferred to individual cages with a running wheel (Actimetrics PT2-MCR2) and *ad libitum* food and water, and entrained to a strict 12hr:12hr light:dark cycle in circadian cabinets (Actimetrics PT2-CCM1 with added running wheel power rails). After allowing at least a week for photoentrainment, wheel-running activity was recorded for at least 10 days in 12:12LD, after which constant darkness was begun at lights off and wheel-running activity was recorded for an additional 15 days. Recording was logged and circadian parameters were analyzed using Clocklab recording and analysis software (Actimetrics).

#### Piezo sleep recordings

Mice were entrained to a strict 12hr:12hr light:dark cycle in circadian cabinets in their home cages for at least a week before beginning the experiment. Mice were then transferred to fresh cages, and placed onto PiezoSleep Mouse Behavioral Tracking System recording platforms.^68,69^ Mice were given at least 3 days to adapt, followed by at least 4 days of recording that were averaged for recording sleep/wake behavior.

#### Tanycyte adult neurogenesis lineage tracing

For lineage tracing of tanycyte-derived adult born cells, tanycytes in Tan^*Bmal1* KO^ mice and littermate controls were labeled through tdTomato expression induced by TAM chow administration for 35 days starting at P30–40. This time period was chosen based on the time it takes for ablation of tanycyte neurogenesis to affect food intake and body mass.^70^ Starting at P65–75, mice were placed back on control 2016 chow for an additional 35 days to allow tanycyte-born cells to achieve a mature cell fate. Animals were then anesthetized with isoflurane before intracardiac perfusion with PBS and 4% PFA (Electron Microscopy Sciences). Brains were fixed in 4% PFA overnight at 4C, and 100μm coronal brain sections sampling the entire hypothalamus were obtained using a vibrating microtome (Leica VT1200S). Brain sections were stained with 1:5000 Hoechst in PBS for 15 min to label cell nuclei before being mounted on microscope slides. Hoechst staining patterns within hypothalamic nuclei were then used to identify brain sections corresponding to bregma −1.35mm, −1.55mm, −1.75mm, and −1.85mm for tanycyte-derived cell counts (Figures 3 and 4). TdTomato^+^ neurons within the arcuate nucleus and all tdTomato^+^ astrocytes within these brain sections were counted for analysis. Astrocytes were distinguished from neurons on the basis of their characteristic “star-like” morphology (Figure 3A). For cell count comparisons between control and Tan^*Bmal1* KO^ mice, female and male mice were each normalized to littermate controls of the same sex.

For neuronal subtype fate mapping experiments, mice were placed on control 2016 chow for 90 days after TAM chow administration to measure the fate acquisition of adult-born feeding neurons corresponding to the 12-week experimental window in which we investigated weight gain on HFD. PFA-perfused brains were fixed in 4% PFA overnight at 4C and then cryoprotected in 30% sucrose in PBS gently rocking overnight at 4C until they were no longer buoyant. Following cryoprotection, 25 μm coronal brain sections were collected on a Leica CM3050 cryostat, with every 5th section mounted onto alternating Superfrost Plus slides (to create multiple subseries sampling the entire hypothalamus).

#### Immunohistochemistry

Fluorescent immunostaining was performed as previously described.^22^ In brief, brains were permeabilized with 1X PBS +0.2% Triton X-100. Antigen retrieval was performed with 10mM sodium citrate buffer for POMC/NPY immunostaining, with slides incubated at 95C for 5 min. Slides were blocked with 5% bovine serum albumin, 10% normal donkey serum, and 0.2% Triton X-100 in 1X PBS for 2 h. Primary antibodies, rabbit anti-POMC (1:5000, Phoenix Pharmaceuticals, Cat# H-029–30, RRID:AB_2307442) and goat anti-NPY (1:200, Novus, Cat# NBP1–46535, RRID:AB_10009813) or rabbit anti-BMAL1 (1:1000, ThermoFisher, Cat# PA1–46118, RRID:AB_2258598) were diluted in blocking solution and slides were incubated overnight at 4C. Sections were washed with 1X PBS and incubated with secondary antibodies Alexa Fluor 647 Donkey Anti-Rabbit IgG (Abcam) and Alexa Fluor 488 Donkey Anti-Goat IgG (Abcam) or Alexa Fluor 488 Donkey Anti-Rabbit IgG (Abcam) diluted 1:250 in blocking solution for 2 h at room temperature. Slides were washed with 1X PBS and stained with 1:1000 Hoechst in 1X PBS for 5 min to label cell nuclei prior to coverslipping with Prolong Diamond antifade mounting media (Fisher). Sections were imaged on Leica STELLARIS 8 confocal microscope (Leica Microsystems) and all immunofluorescent analyses were performed blinded and using ImageJ (NIH).

### QUANTIFICATION AND STATISTICAL ANALYSIS

#### Sequencing analysis

For our analysis single cell RNA-Seq data from,^24^ we selected cells annotated as “Tanycyte1″ and “Tanycyte2″ for our tanycyte analysis and cells annotated as “Ependymo” for our ependymocyte analysis. Differential expression analyses were performed between the high fat diet group (HFD) and the control chow group (Ch10) using the Seurat function FindMarkers with default parameters. We then filtered for significant genes using the thresholds adjusted *p*-value <0.2 and absolute log_2-fold change >0.25. Finally, we performed pathway analyses on the significantly DE genes using the function DEenrichRPlot on databases “GO_Biological_Process_2023″ and “GO_Molecular_Function_2023”.

#### Statistics

GraphPad Prism 10.0 was used for statistical analyses. For comparisons between two experimental groups, unpaired two-tailed Student’s t tests were used. For comparisons between more than two experimental groups, a two-way ANOVA test with a Holm-Sidak multiple-comparison post hoc analysis was performed to compare the differences between individual groups. For comparisons between more than two experimental groups over time, a repeated measures 3-way ANOVA test was used. A *p* value of less than 0.05 was considered statistically significant. The statistical tests, n (number of animals), and *p* values for each dataset are provided in the figure legend that accompanies the data. Experiments were performed and quantified by investigators who were blinded to the genotype and treatment of the mice, and were unblinded once summary data were ready to be prepared.

## Supplementary Material

1

SUPPLEMENTAL INFORMATION

Supplemental information can be found online at https://doi.org/10.1016/j.celrep.2026.117685.

## Figures and Tables

**Figure 1.**
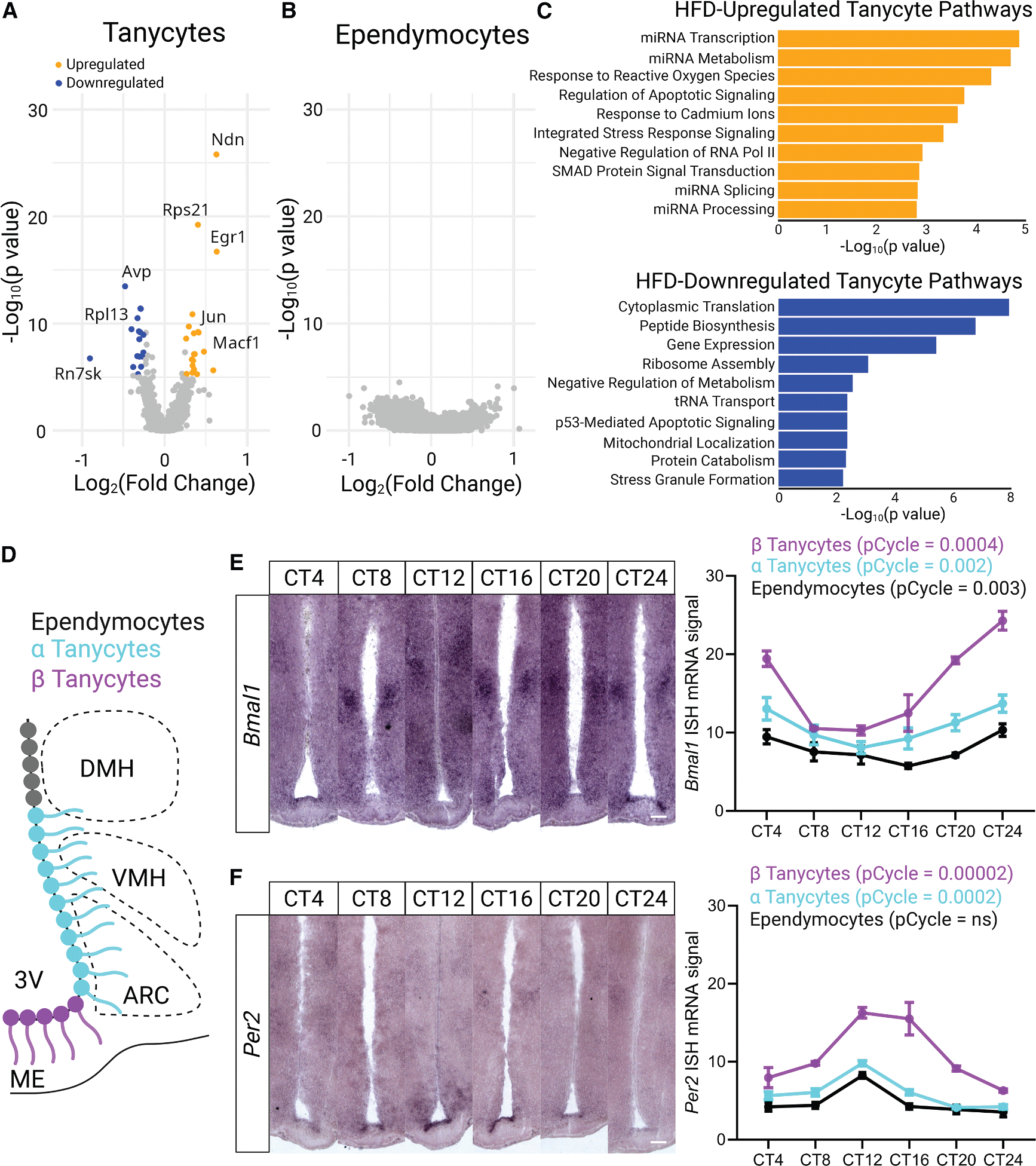
Diet-induced gene expression and circadian oscillation of core clock genes in tanycytes and ependymocytes (A and B) Volcano plots showing differentially expressed genes (DEGs) in tanycytes (A) and ependymocytes (B) from mice fed HFD vs. LFD for 1 week (dataset from.^24^ (C) Pathway analysis of significantly diet-regulated tanycyte DEGs from (A) using Gene Ontology enrichment analysis. (D) Coronal diagram of mouse hypothalamus indicates cell populations spanning the third ventricle. (E–F **left**) Representative *in situ* hybridization (ISH) images of *Bmal1* (B) and *Per2* (C) mRNA in tanycytes and ependymocytes across circadian time (CT; *n* = 3–4 mice/CT). Scale bars, 150 μm. (E and F **right)** ISH quantification. Mean ± SEM, tested for significant circadian rhythmicity (pCycle) by JTK-cycle, ns = not significant. To understand the potential impact of HFD on tanycyte function, we performed a literature search on differentially expressed genes from this experiment. Prior literature on highly up-regulated individual transcripts (e.g., *Necdin*, *Rps21, Egr1*) suggested an inhibitory effect of dietary fat on hypothalamic neurogenesis, consistent with HFD effects on ARC but not ME neurogenesis.^20,21^ Both *Necdin* and *Rps21* are anti-proliferative,^25,26^ and Egr1 stimulates IGF receptor signaling, which specifically inhibits tanycyte neurogenesis and self-renewal.^18,27^ Members of the senescence-promoting AP-1 complex including *Jun* and *FosB* were also up-regulated.^28^ Several transcripts are also associated with aspects of neuronal differentiation, including *Necdin* (differentiation and survival of postmitotic neurons),^26^ *Macf1* (neurite outgrowth and migration),^29^ *Jun* (associated with post-mitotic neurons more than neural precursors in adult neurogenesis),^30^ and *Rps21* (most enriched ribosomal factor in dendrites).^31^ Consistent with a potential anti-proliferative effect of HFD in tanycytes, Gene Ontology analysis identified changes in cellular pathways associated with ATF4 signaling, including the upregulation of reactive oxygen species-associated pathways and the integrated stress response as well as the downregulation of cytoplasmic translation and ribosome assembly (Figure 1C).^32,33^

**Figure 2.**
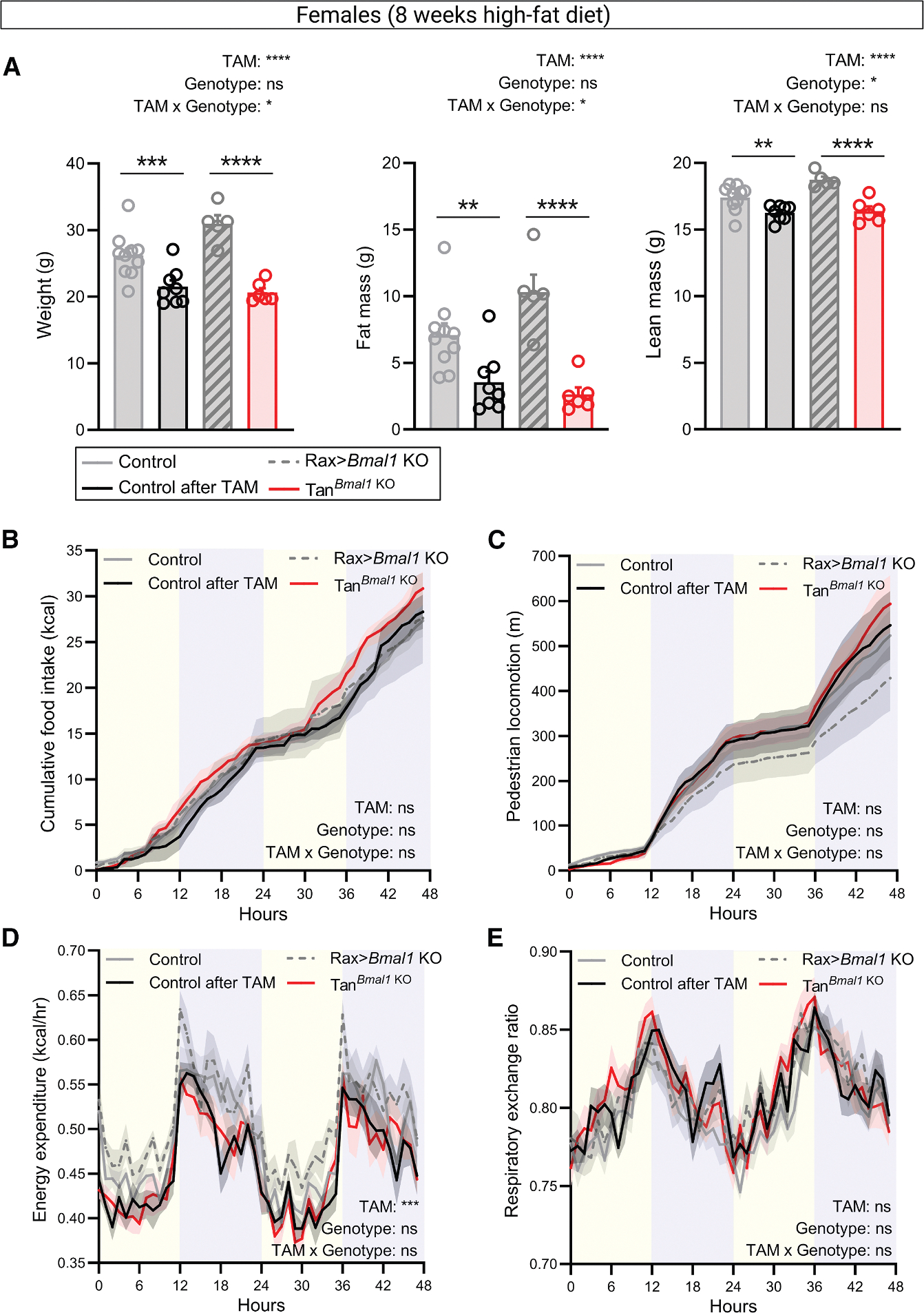
Adult tanycyte *Bmal1* KO reduces weight gain in female mice on HFD (A) Weight (left), fat mass (middle), and lean mass (right) of female mice following metabolic monitoring after 8 weeks on HFD (*n* = 5–10 mice/group). (B–E) Metabolic monitoring data for female mice for 48 h after 8 weeks of HFD including cumulative food intake (B, *n* = 4–7 mice/group), pedestrian locomotion (C, *n* = 5–10 mice/group), energy expenditure (D, *n* = 5–9 mice/group), and respiratory exchange ratio (E, *n* = 5–9 mice/group). Mean ± SEM, *p < 0.05, **p < 0.01, ***p < 0.001, and ****p < 0.0001, ns = not significant, repeated measures 3-way ANOVA for time series data, two-way ANOVA with Holm-Sidak post hoc test for weight and MRI data.

**Figure 3.**
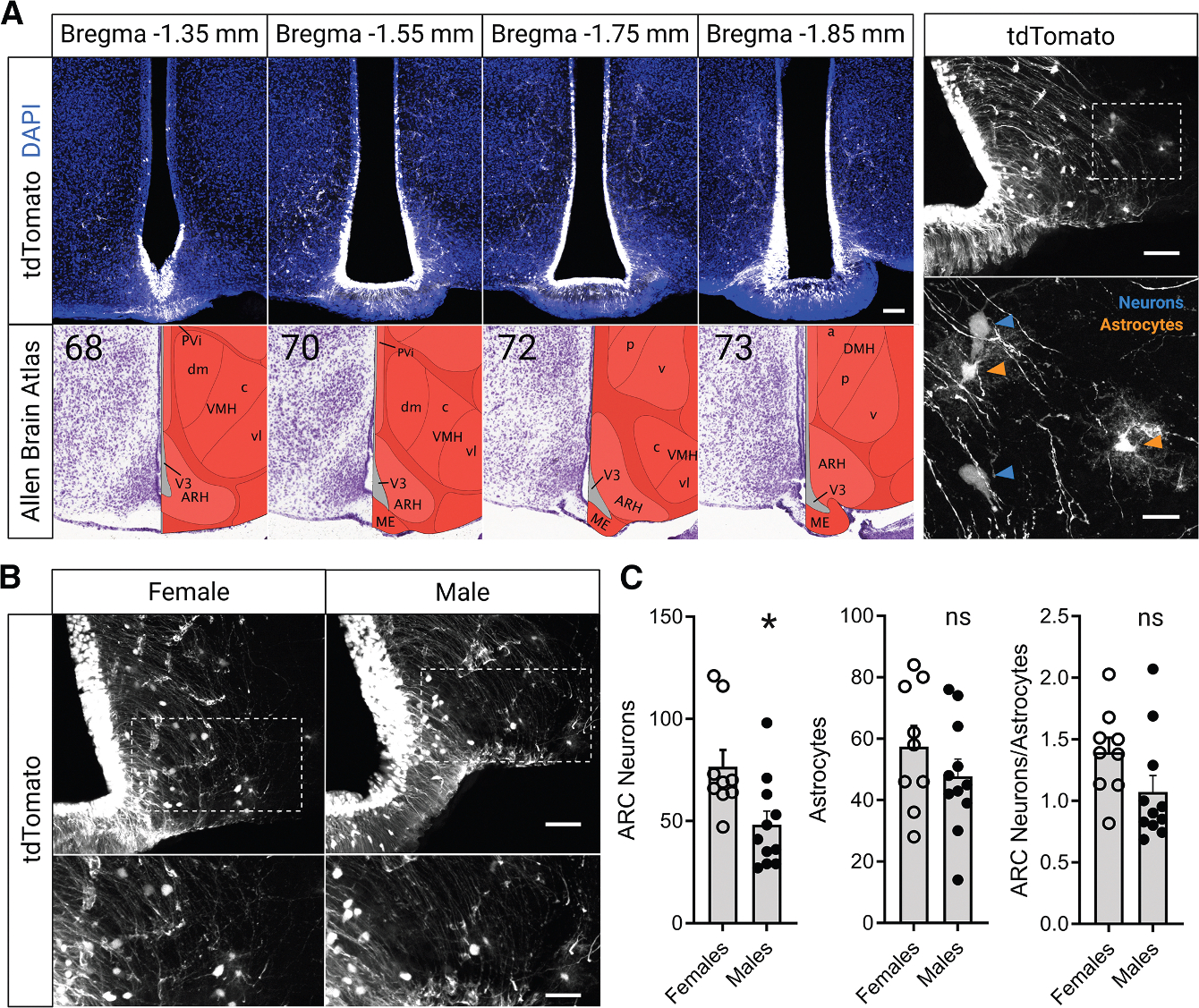
Lineage tracing reveals that levels of tanycyte adult neurogenesis are sex-specific (A) Left: representative 100μm hypothalamus slices used for lineage tracing cell counts (top), matched to corresponding Allen Brain Atlas images (bottom). Scale bars, 100μm. Right: representative slice (top) and magnified region of interest (bottom) showing tanycyte-derived ARC neurons (blue arrows) and astrocytes (orange arrows). Scale bars, 100, 75μm. (B) Representative coronal slices (top) and magnified regions of interest (bottom) of ARC neurons and astrocytes born from tanycytes in female and male mice after 35 days of control chow following TAM treatment. Scale bars, 100, 75 μm. (C) Quantification of adult-born ARC neurons (left), astrocytes (middle), and neuron/astrocyte ratio derived from tanycytes in female and male mice from 4 coronal tissue sections corresponding to Bregma coordinates from 3A (*n* = 9–11 mice/group). Mean + SEM. *p < 0.05, ns = not significant, Student’s *t* test.

**Figure 4.**
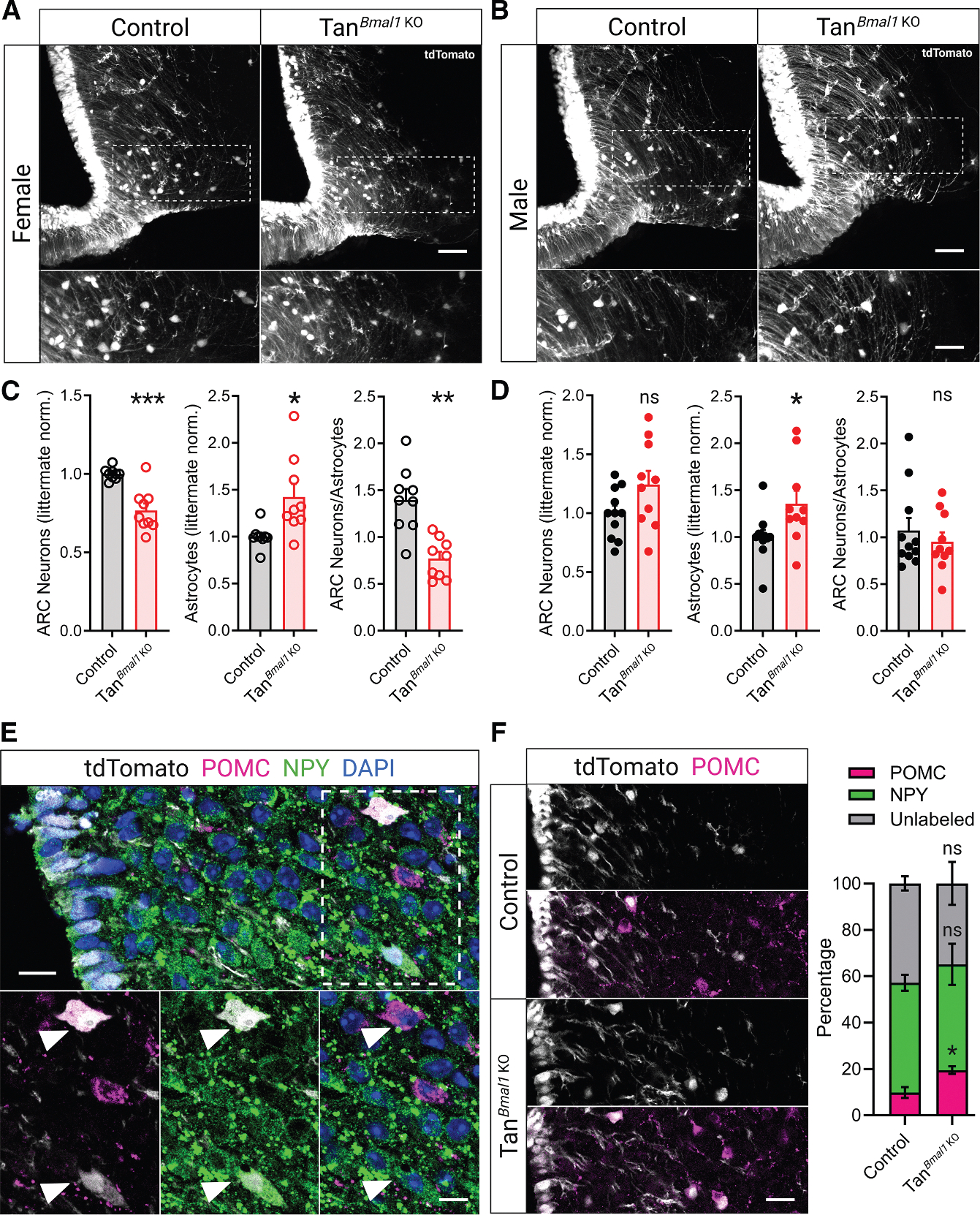
Impact of *Bmal1* KO on tanycyte neurogenesis is sex-specific (A and B) Representative coronal sections (top) and magnified regions of interest (bottom) show adult-born ARC neurons and astrocytes derived from tanycytes in control and Tan^*Bmal1* KO^ mice after 35 days of control chow following TAM treatment. Scale bars, 100, 75 μm. (C and D) Quantification of adult-born ARC neurons (left), astrocytes (middle), and neuron/astrocyte ratio (right) of tanycyte-derived cells from control and Tan^*Bmal1* KO^ mice from 4 coronal tissue sections corresponding to Bregma coordinates from 3A (*n* = 9 female and 10–11 male mice/group). (E) Representative z-planes (top) and magnified regions of interest (bottom) with white arrows indicating POMC^+^ satiety (magenta) and NPY^+^ hunger (green) neurons lineage traced from tanycytes (white). Blue indicates Hoechst^+^ nuclei. Scale bars, 30 μm. (F) Representative z-planes (left) and quantification (right) of tanycyte-derived ARC neuron fates in control and Tan^*Bmal1* KO^ mice after 12 weeks on control chow (*n* = 4–5 female mice/group). Scale bars, 50 μm. Mean ± SEM, *p < 0.05, **p < 0.01, and ***p < 0.001, ns = not significant, *p < 0.05, Student’s *t* test.

**KEY RESOURCES TABLE:** 

REAGENT or RESOURCE	SOURCE	IDENTIFIER

Antibodies		

Rabbit anti-POMC	Phoenix Pharmaceuticals	Cat# H-029-30; RRID: AB_2307442
Goat anti-NPY	Novus	Cat# NBP1-46535; RRID: AB_10009813
Rabbit anti-BMAL1	Thermo Fisher Scientific	Cat# PA1-46118; RRID: AB_2258598
Alexa Fluor^®^ 647 Donkey Anti-Rabbit IgG	Thermo Fisher Scientific	Cat# A-31573; RRID: AB_2536183
Alexa Fluor^®^ 488 Donkey Anti-Goat IgG	Thermo Fisher Scientific	Cat# A-11055; RRID: AB_2534102
Alexa Fluor^®^ 488 Donkey Anti-Rabbit IgG	Abcam	Cat# A32790; RRID: AB_2762833

Chemicals, peptides, and recombinant proteins		

Hoechst	Life Tech	Cat# 62249

Experimental models: organisms/strains		

B6.129S4(Cg)-*Bmal1^tm1Weit^*/J (Bmal1 floxed allele)	The Jackson Laboratory	RRID: IMSR_JAX:007668
B6.129-*Bmal1^tm1Bra^*/J (*Bmal1* constitutive null allele)	The Jackson Laboratory	RRID: IMSR_JAX:009100
B6.Cg-*Gt(ROSA)26Sor^tm14(CAG-tdTomato)Hze^*/J (Ai14 fluorescent reporter)	The Jackson Laboratory	RRID: IMSR_JAX:007914
*Rax^tm1.1(cre/ERT2)Sbls^*/J (*RaxCreER*)	Seth Blackshaw - Johns Hopkins University School of Medicine	RRID: IMSR_JAX:025521
C57BL/6J	The Jackson Laboratory	RRID: IMSR_JAX:000664

Oligonucleotides		

*Bmal1* Exon 8 forward primer: ATGCAGAACACCAAGGAAGG	Integrated DNA Technologies (IDT)	N/A
*Bmal1* Exon 8 reverse primer: CTTCCTCGGTCACATCCTA	Integrated DNA Technologies (IDT)	N/A

Recombinant DNA		

*Per2* Plasmid	Seth Blackshaw - Johns Hopkins University School of Medicine	Accession #AI838843
*Bmal1* Plasmid (pCRII-TOPO backbone)	This paper	N/A

Software and algorithms		

Prism 10 GraphPad	https://www.graphpad.com/scientific-software/prism/	RRID: SCR_002798
FIJI (ImageJ)	NIH	RRID: SCR_002285
JTK_CYCLE	Hughes et al.^59^	RRID: SCR_017962
MetaCycle R	Wu et al.^60^	RRID: SCR_025729
Clocklab	Actimetrics	RRID: SCR_014309
PiezoSleep Mouse Behavioral Tracking System	Signal Solutions	N/A
CalR2	Mina et al.^61^; https://calrapp.org/	RRID: SCR_015849

Other		

Tamoxifen chow	Inotiv/Envigo	TD.130856
High-fat diet (60% calories from fat)	Inotiv/Envigo	TD.06414
Control 2016 chow	Inotiv/Envigo	2916.cs

## Highlights

- Tanycytes show circadian rhythms of clock gene expression
- Adult, tanycyte-specific *Bmal1* knockout reduces diet-associated weight gain in female mice
- Lineage tracing shows that female mice produce more tanycyte-born arcuate neurons than males
- *Bmal1* knockout reduces tanycyte neurogenesis in female but not male mice
