# Supplementary material for: Tanycyte BMAL1 regulates high-fat diet weight gain and shapes arcuate neurogenesis in female mice
Source: Cell Rep. Author manuscript; Available in PMC 2026 Aug 13. (PMC13470997; doi:10.1016/j.celrep.2026.117685)
Supplement: Supplementary file 1 [file NIHMS2199977-supplement-1.pdf]

Cell Reports, Volume *45*

Supplemental information

Tanycyte BMAL1 regulates high-fat diet weight gain and shapes arcuate neurogenesis in female mice

Daniel Maxim Iascone, Pavel Pivarshev, Jianing Yang, Mariela Lopez Valencia, Sara B. Noya, Hongtong Lin, Corey D. Holman, Ron C. Anafi, Joseph L. Bedont, and Amita Sehgal

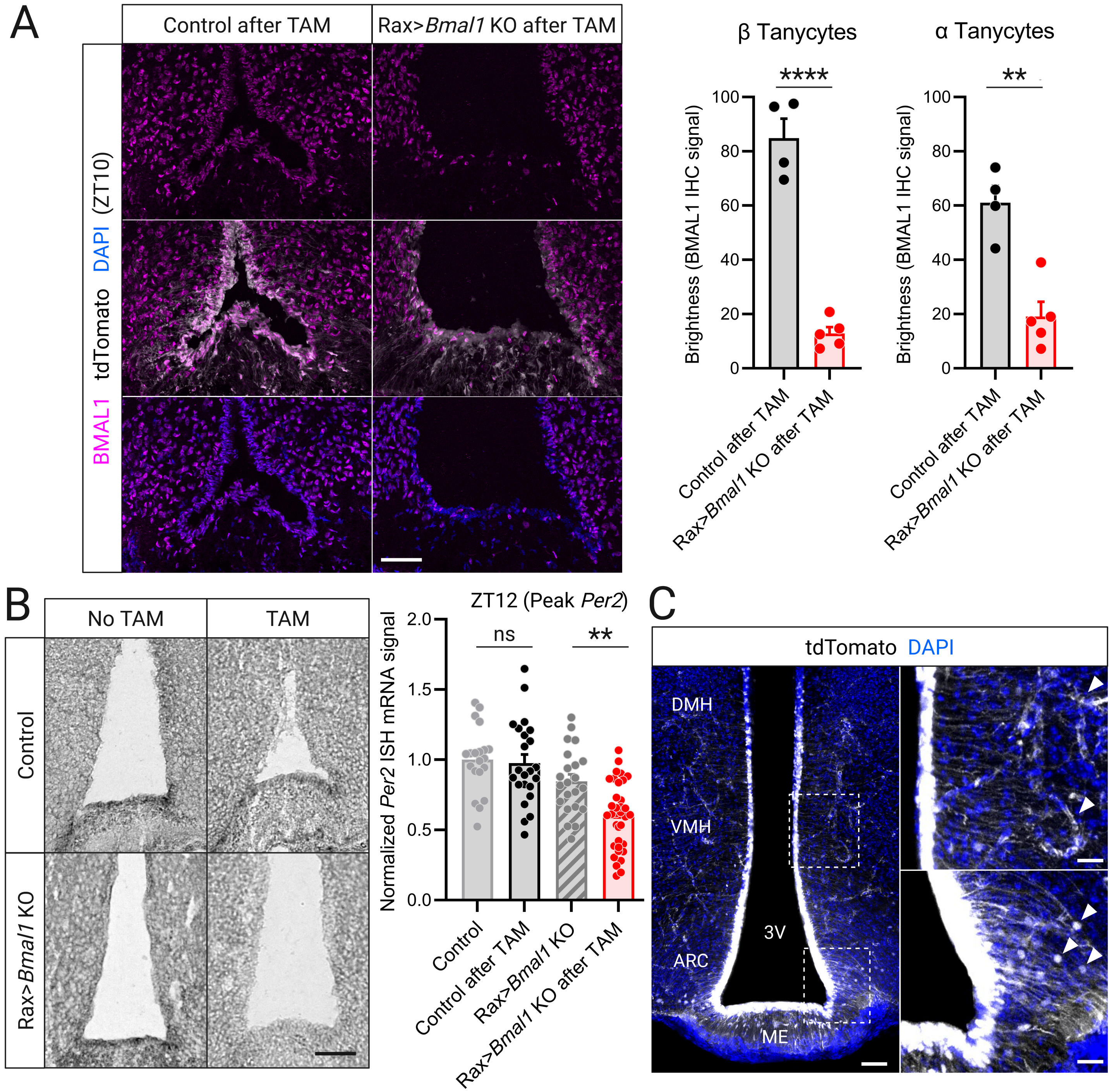

Supplementary Figure 1. Tanycyte-specific *Bmal1* KO and lineage tracing with *RaxCreER* driver mice
(**A**) Representative images (left) and quantification (right) of BMAL1 immunostaining at ZT10 demonstrating reduced expression in tdTomato$^+$ tanycytes of Tan$^{Bmal1\ KO}$ mice after 35 days of control chow following TAM treatment. (n = 4-5 mixed sex mice per group). Scale bar = 100 µm. (**B**) Representative images (left) and quantification (right) of *Per2* mRNA levels at ZT12 in mice following 12 weeks of HFD demonstrates that tanycyte-specific *Bmal1* KO through tamoxifen (TAM)-induced recombination in *RaxCreER* mice reduces peak *Per2* expression (same mice as in Figure 3; n = 20-35 mixed-sex mice per group). Scale bar = 150 µm. (**C**) Coronal brain section of mouse hypothalamus 35 days following RaxCreER-driven tdTomato expression (white; same image as Figure 3A panel 3 with magnified regions of interest for detailed examination). Dashed boxes indicate magnified regions with tanycyte processes contacting blood vessels (white arrows in top region of interest) and tanycyte-born neurons (white arrows in bottom region of interest). Blue indicates Hoechst$^+$ nuclei. 3V third ventricle, ARC arcuate nucleus, DMH dorsomedial nucleus, ME median eminence, VMH ventromedial nucleus. Scale bars = 50µm (left), 20µm (right). Mean + SEM, **p<0.01, ****p<0.0001, ns = not significant, Student's t-test for immunostaining data, 2-way ANOVA with Holm–Sidak post hoc test for ISH data.

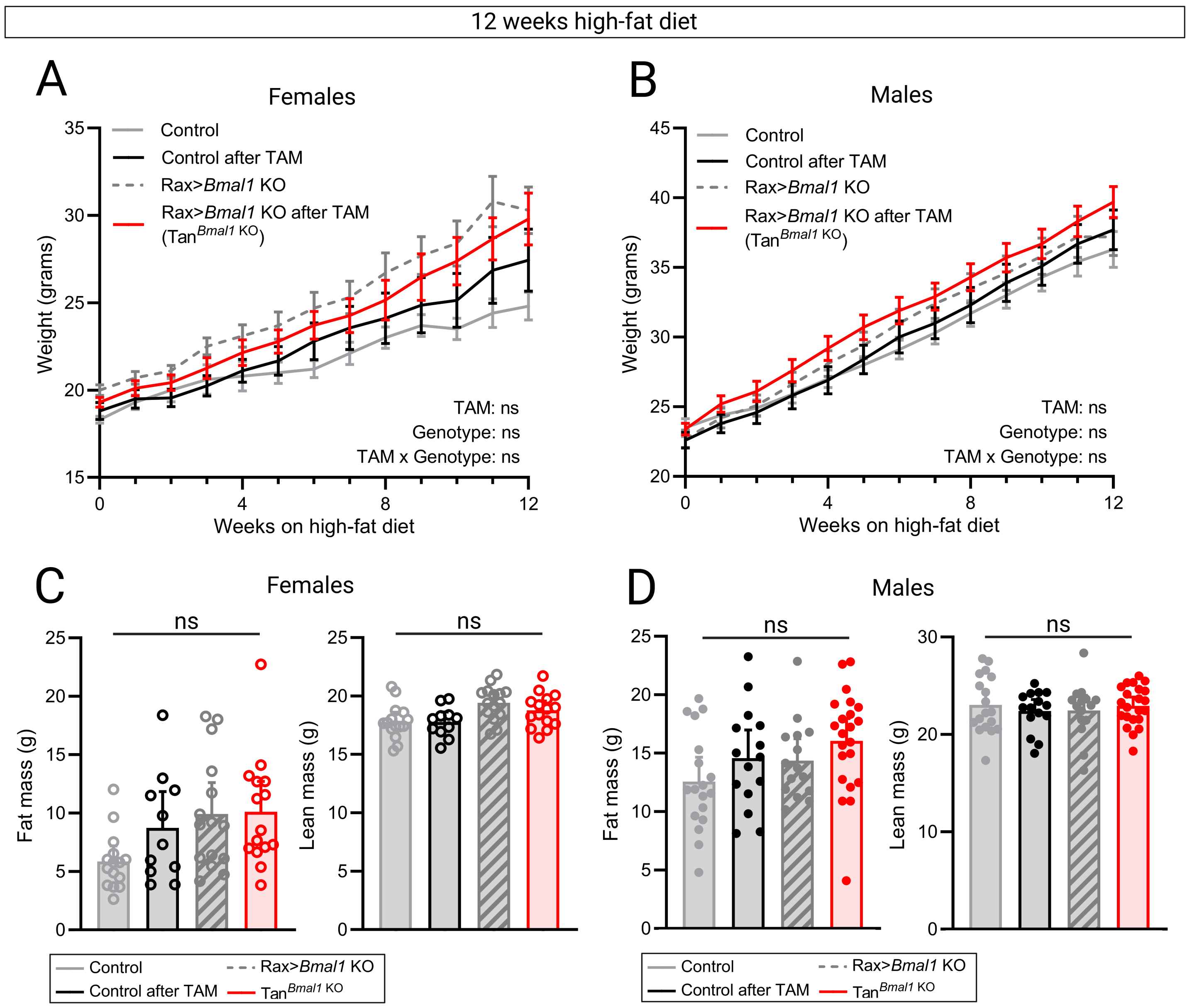

Supplementary Figure 2. Weight gain and body composition is not altered in Tanycyte *Bmal1* KO mice following 12 weeks of high-fat diet

(**A-B)** Weight gain on HFD in female and male mice (n = 10-15 female and 15-22 male mice/group). (**C-D**) Fat mass (left) and lean mass (right) measured with MRI of female (C) and male (D) mice following 12 weeks of HFD (n = 11-15 female and 15-22 male mice per group). Error bars indicate mean + SEM. ns = not significant, repeated measures 3-way ANOVA for time series data and 2-way ANOVA with Holm–Sidak post hoc test for MRI data.

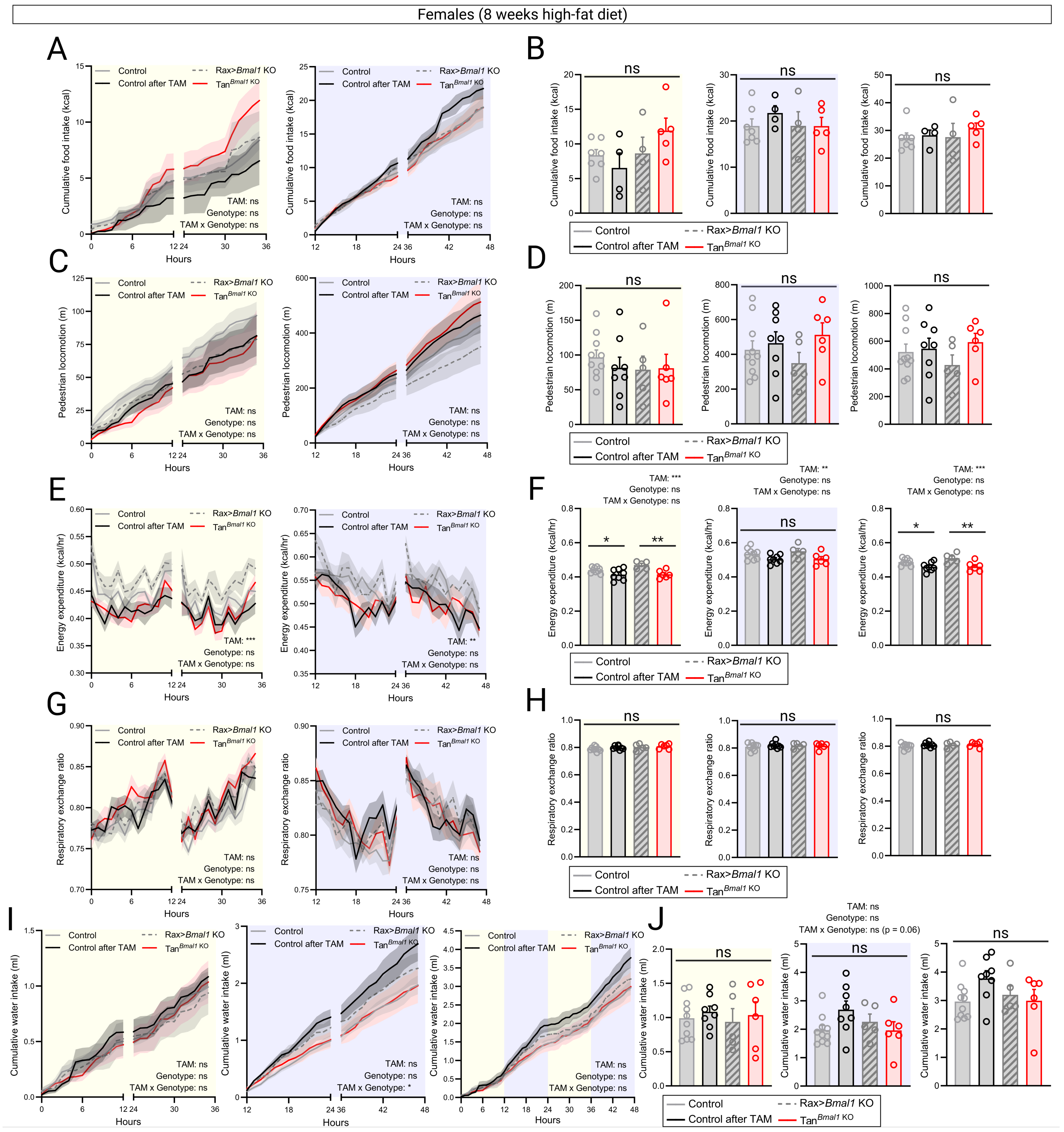

Supplementary Figure 3. Light period and dark period splits for metabolic monitoring experiments
(**A**) Cumulative food intake for female mice for 48 hours after 8 weeks of HFD binned into light periods (yellow) and dark periods (blue; n = 4-7 mice/group). (**B**) Food intake totals from A for light periods (left), dark periods (middle), and total monitoring window (right). (**C**) Cumulative locomotion for female mice for 48 hours after 8 weeks of HFD binned into light and dark periods (n = 5-10 mice/group). (**D**) Locomotion totals from C for light periods (left), dark periods (middle), and total monitoring window (right). (**E**) Energy expenditure for female mice for 48 hours after 8 weeks of HFD binned into light and dark periods (n = 5-9 mice/group). (**F**) Average energy expenditure from E for light periods (left), dark periods (middle), and total monitoring window (right). (**G**) Respiratory exchange ratios for female mice for 48 hours after 8 weeks of HFD binned into light dark periods (n = 5-9 mice/group). (**H**) Average respiratory exchange ratios from G for light periods (left), dark periods (middle), and total monitoring window (right).

(**I**) Cumulative water intake female mice for 48 hours after 8 weeks of HFD binned into light periods (left) and dark periods (middle), and total monitoring window (right; n = 5-10 mice/group). (**J**) Cumulative water intake from I for light periods (left), dark periods (middle), and total monitoring window (right). Mean ± SEM, *p<0.05, **p<0.01, ***p<0.001, ns = not significant, repeated measures 3-way ANOVA for time series data, 2-way ANOVA with Holm–Sidak post hoc test for total and average data. Main and interaction effects of genotype and TAM are not significant unless otherwise indicated.

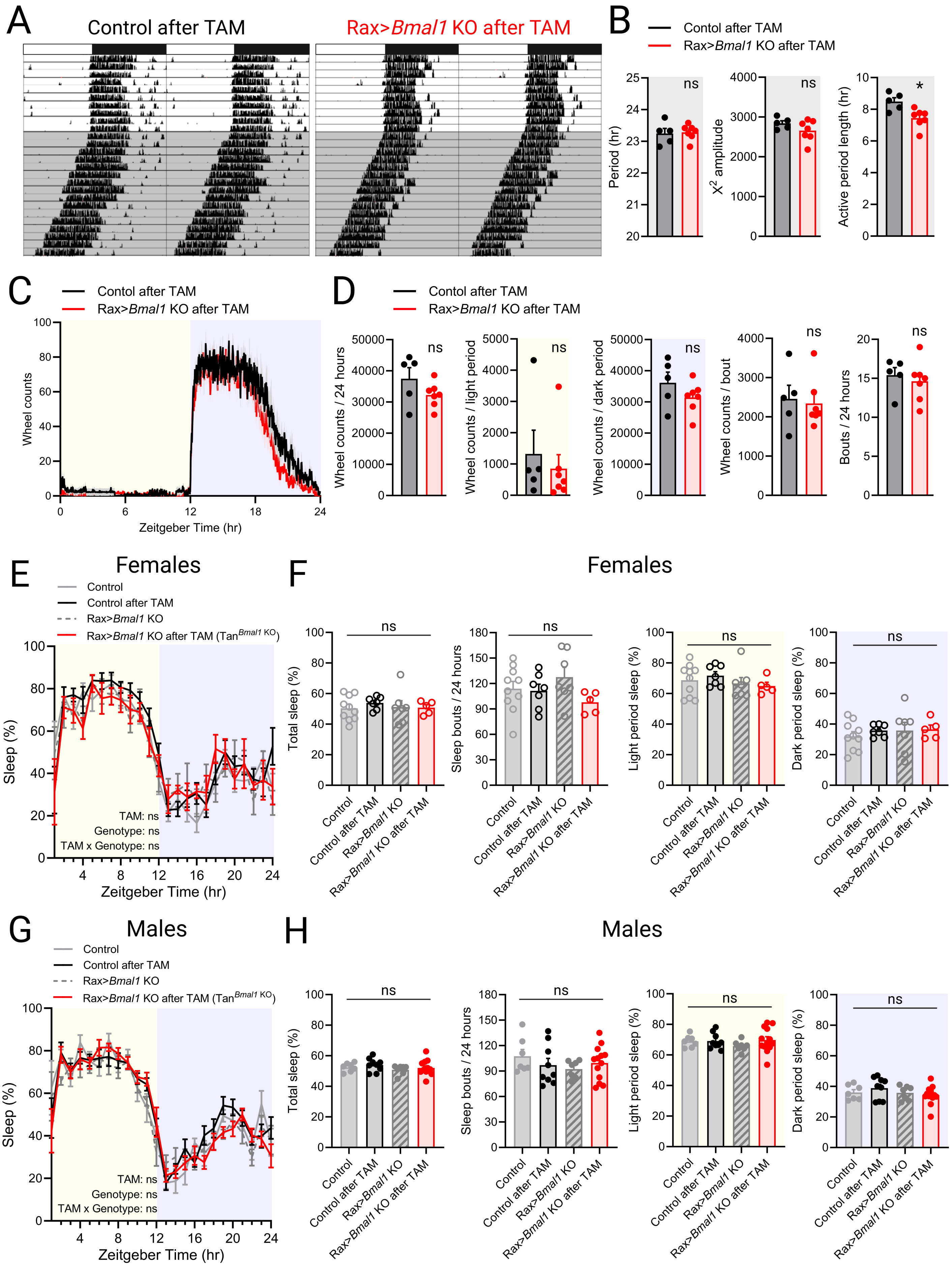
A
Control after TAM
Rax>Bmal1 KO after TAM
B
Contol after TAM
Rax>Bmal1 KO after TAM
Period (hr)
X2 amplitude
Active period length (hr)
ns
ns
*
C
Contol after TAM
Rax>Bmal1 KO after TAM
Wheel counts
Zeitgeber Time (hr)
D
Contol after TAM
Rax>Bmal1 KO after TAM
Wheel counts / 24 hours
Wheel counts / light period
Wheel counts / dark period
Wheel counts / bout
Bouts / 24 hours
E
Females
Control
Control after TAM
Rax>Bmal1 KO
Rax>Bmal1 KO after TAM (TanBmal1 KO)
Sleep (%)
TAM: ns
Genotype: ns
TAM x Genotype: ns
Zeitgeber Time (hr)
F
Females
Total sleep (%)
Sleep bouts / 24 hours
Light period sleep (%)
Dark period sleep (%)
Control
Control after TAM
Rax>Bmal1 KO
Rax>Bmal1 KO after TAM
G
Males
Control
Control after TAM
Rax>Bmal1 KO
Rax>Bmal1 KO after TAM (TanBmal1 KO)
Sleep (%)
TAM: ns
Genotype: ns
TAM x Genotype: ns
Zeitgeber Time (hr)
H
Males
Total sleep (%)
Sleep bouts / 24 hours
Light period sleep (%)
Dark period sleep (%)

Supplementary Figure 4. Tanycyte *Bmal1* KO does not alter circadian period, total locomotor activity, or sleep (**A**) Representative double-plotted running wheel actograms for control and Tan$^{Bmal1\ KO}$ mice after TAM. White and black bars denote 12:12-hour LD cycle for the first 10 days and the gray shaded area denotes DD (last 15 days) on control chow. (**B**) Average circadian period (left), χ^2 amplitude (middle), and active period length (right) of wheel-running activity during 15 days of constant darkness (n = 5-7 mixed-sex mice per group). Gray graph shading: constant darkness. (**C**) Wheel counts per minute across the 24-hour day of mice housed in a 12:12-hour LD cycle during the first 10 days of the experiment (n = 5-7 mixed-sex mice per group). Yellow graph shading: light period; blue graph shading: dark period. (**D**) Left to right: wheel counts per 24 hours, per light period, per dark period, per bout, and bouts per 24 hours of wheel-running from (C). (**E**) Percent time asleep in female mice binned in 1-hour intervals averaged across 4 days on control chow (n = 5-10 mice/group). (**F**) From left to right: percent time asleep, sleep bouts/day, percent time spent asleep during the light period, and percent time asleep during the dark period of female mice quantified from E. (**G**) Same as E for male mice 7-13 mice/group). (**H**) Same as F for male mice quantified from G. Mean ± SEM. *p<0.05, ns = not significant, Student's t-test (B, D), repeated measures 3-way ANOVA for time series data, and 2-way ANOVA with Holm–Sidak post hoc test (F, H).
